# Analysis of correlations between zona pellucida birefringence and molecular markers of oocyte developmental competence

**DOI:** 10.1186/1471-2164-15-S2-P48

**Published:** 2014-04-02

**Authors:** Mourad Assidi, Markus Montag, Marc-André Sirard

**Affiliations:** 1 Centre de Recherche en Biologie de la Reproduction, Laval University, Quebec City, QC, G1K 7P4, Canada; 2 Center of Excellence in Genomic Medicine Research, King Abdulaziz University, Jeddah, 21589, Saudi Arabia; 3 KACST Technology Innovation Center in Personalized Medicine, King Abdulaziz University, Jeddah, 21589 Saudi Arabia; 4 Department of Gynecological Endocrinology and Reproductive Medicine, Bonn University, Bonn, Germany

## Background

Human infertility is the incapacity of a couple to conceive after one year of unprotected sexual intercourse. Selection of the best gametes for subsequent steps of fertilization and embryo transfer was shown to be the crucial step in infertility treatment procedure [1,2]. Oocyte selection using morphological criteria has been the gold standard method in assisted reproductive technologies (ART) clinics. Zona Pellucida (ZP) , a filamentous matrix of glycosylated glycoproteins surrounding the oocyte, is one of these morphological criteria of oocyte selection. In fact, ZP thickness and birefringence was reported to be positively correlated with higher ability of the oocyte to achieve successful pregnancy, but this selection approach has limitations in terms of accuracy, objectivity and constancy. Recent studies using OMICs approaches have identified key molecular markers in somatic cells (cumulus and/or granulosa cells) and follicular fluid that quantitatively and non-invasively predict the oocyte quality for better selection, higher pregnancy rates and efficient infertility treatment. These biomarkers could be a valuable reinforcement of the morphological selection criteria widely used in IVF clinics. In this context, this study was designed to study the relationship between some molecular predictors of oocyte quality found by our group and the conventional morphological parameters of oocyte quality. We expect to find a positive correlation between the ZP birefringence and molecular markers of oocyte competence. Such integrative strategy should lead to a powerful combined approach that will precisely predict the oocyte developmental potential, allowing therefore efficient infertility treatment and elective single embryo transfer (eSET).

## Materials and methods

**Figure 1:**
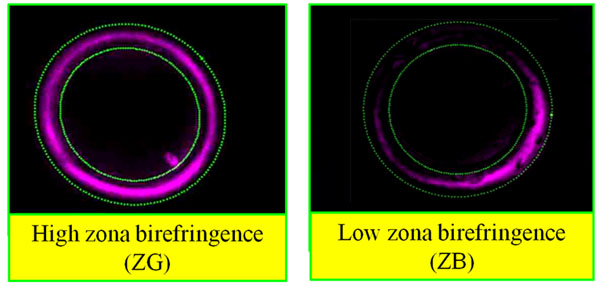
Automatic scoring at 180 points of ZP of MII oocytes using the Octax PolarAIDE polarized microscopy (MTG 2010)

Seven (7) patients with informed consent were selected for ovarian stimulation, ICSI (intracytoplasmic sperm injection) and subsequent embryo transfer at day 3 at the IVF clinic of Bonn University Medical School. Cumulus-oocyte complex (COC) were retrieved 36 to 38 h after HCG administration and washed. While cumulus cells (CCs) of each oocyte were individually collected and put at -80°C for subsequent RNA extraction (PicoPure, Molecular Devices, CA), the metaphase II (MII) oocytes were completely denuded by hyaluronidase followed successively by automatic ZP birefringence assessment (Octax polairAide™, OCTAX Microscience GmbH, Altdorf, Germany); [3]) and ICSI one hour later. MII oocytes having an inner zona layer with reduced or asymmetrical birefringence were considered as Zona Bad (ZB). Conversely, oocytes with high and uniform birefringence were named Zona good (G)[3,4] (Figure 1). After ICSI and individual culture, the two embryos having the two top ZP birefringence scores were transferred. Successful pregnancy evaluated by HCG test at day 14, and confirmed by ultrasons at week 5. Both ZP birefringence and pregnancy results were used to distinguish two groups of patients 1) High ZP birefringence and successful pregnancy (ZGP), versus 2) Low/irregular ZP birefringence and pregnancy failure (ZBNP). After RNA amplification (RiboAmpplus RNA Amplification kit, Molecular Devices), differentially expressed gene analysis in CCs of ZGP (8 CCs samples) versus ZBNP (3 CCs samples) was done by hybridization using two platforms: a custom- made microarray [5-7] as well as the OneArray chips (30K; Phalanx Biotech, Palo Alto, CA). Data analysis using National Institute on Aging (NIA) Array Analysis Tool (Baltimore, MD) [8] allowed the identification of both over-expressed and under-expressed gene lists (fold change ≥ 2; FDR=5%) of the ratio ZGP/ZBNP; and the identification of reliable markers of oocyte quality associated to ZP birefringence and pregnancy output. Selected gene markers were validated by QPCR.

## Results

**Figure 2:**
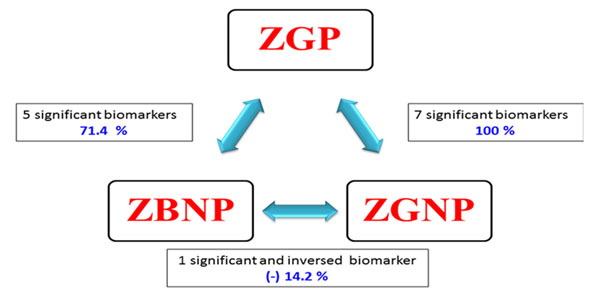
Schematic representation of the correlation (expressed in %) of significant gene biomarkers between the three morphological groups of ZB

Possible correlation between 7 biomarkers differentially expressed in the CCs of good quality oocytes [9] and the ZP birefringence was analyzed in order to assess if these two approaches (combination of ZP morphology and molecular markers) are additive, and hence able to strengthen the highly competent oocytes selection procedure. 32 and 50 candidate genes were respectively under-expressed and overexpressed in the ZGP compared to the ZBNP. Interestingly, three (3) positive biomarkers from the (ZGP vs ZGNP) comparison were also present in the overexpressed gene list of the (ZGP vs ZBNP). These biomarkers are PSMD6, CALM1 and NRP1. Surprisingly, this study demonstrated that most of the analyzed biomarkers were not significantly different between the two groups with failed pregnancy (ZGNP vs ZBNP) (Figure 2). Therefore and despite having two opposite ZP birefringence, few or no transcriptional differences were found between these two groups.

## Conclusions

Weak correlations between the 7 gene biomarkers of oocyte developmental potential and the ZP birefringence score. It looks that the ZP morphology is associated to a transcriptomic gene pattern that is not directly related to the developmental competence pathway. Further studies using larger lists of candidate markers are required to identify suitable genes that are highly correlated with the morphological criteria, and therefore able to reinforce the accuracy of oocyte selection. Together, the combination of these two approaches should offer a reliable prognostic tool of the best oocyte allowing efficient infertility treatment and successful pregnancy.
